# Transarterial chemoembolization combined with iodine 125 seeds versus transarterial chemoembolization combined with radiofrequency ablation in the treatment of early- and intermediate-stage hepatocellular carcinoma

**DOI:** 10.1186/s12876-020-01355-3

**Published:** 2020-06-29

**Authors:** Lei Chen, Xuefeng Kan, Tao Sun, Yanqiao Ren, Yanyan Cao, Liangliang Yan, Bin Liang, Bin Xiong, Chuansheng Zheng

**Affiliations:** 1 Department of Radiology, Union Hospital, Tongji Medical College, Huazhong University of Science and Technology, Wuhan, 430022 China; 2 Hubei Province Key Laboratory of Molecular Imaging, Wuhan, 430022 China

**Keywords:** Hepatocellular carcinoma, Transarterial chemoembolization, Iodine 125, Radiofrequency ablation, Propensity score matching

## Abstract

**Background:**

Transarterial chemoembolization (TACE), radiofrequency ablation (RFA), and iodine 125 seeds implantation are optional treatments for hepatocellular carcinoma (HCC). The aim of this study is to compare the efficacy of the combined treatment of TACE with iodine 125 seeds implantation (TACE-iodine 125) with TACE with RFA (TACE-RFA) in patients with early- and intermediate-stage HCC.

**Methods:**

The study included 112 patients who were diagnosed with early- and intermediate-stage HCC from January 1, 2014, to May 31, 2018. Among them, 38 patients were treated with TACE-Iodine 125, and 74 with TACE-RFA. The efficacy of the two treatment groups was retrospectively analyzed. To reduced the selective bias, a propensity score matching (PSM) analysis and inverse probability of treatment weighting (IPTW) method were used to compare the outcomes between the two groups.

**Results:**

In the absence of PSM and IPTW, the median overall survival (OS) and progression-free survival (PFS) of the TACE-RFA group were slightly longer than those of the TACE-Iodine 125 group (OS: 41 months vs. 36 months; PFS: 18 months vs. 15 months). However, there were no statistically significant differences in the median OS, PFS, and objective response rate (ORR) between the two groups (*P* > 0.05). After adjusting the age, gender, Child-Pugh class, Barcelona Clinic Liver Cancer (BCLC) stage, and Alpha-fetoprotein (AFP), TACE-Iodine 125 treatment was not associated with a significant increasing the risks of death (HR: 0.763; 95%CI: 0.403,1.345, *P* = 0.320) and recurrence (HR: 1.020; 95%CI: 0.645,1.611, *P* = 0.934). After PSM, 35 matched pairs of patients were obtained, and there were no statistically significant differences in the median OS and PFS between the two groups. After IPTW, similar results presented.

**Conclusions:**

The combination of TACE with iodine 125 seeds implantation may represent an effective treatment for patients with early- and intermediate-stage HCC.

## Background

Hepatocellular carcinoma (HCC) is one of the deadliest cancers [1]. The recommended first-line treatments for patients with early stage HCC include surgical resection, liver transplantation, or ablation, while transarterial embolization (TACE) is the treatment of choice for patients in intermediate stage of the disease [2]. In China, the application of liver transplantation is limited due to the high cost and shortage of organs. The efficacy of radiofrequency ablation (RFA) for HCC is similar to that of surgical resection when the diameter of the malignant liver nodule does not exceed 3-cm [3, 4]. However, for medium-to-larger tumors, a fraction of cancer cells may still survive after RFA due to the heat-sink effect [5], that diminishes the efficacy of RFA. Therefore, some combined therapies were used to treat patients with tumors larger than 3-cm. It has been reported that patients with early or intermediate stage HCC had a better survival with the combined treatment of TACE-RFA than with a single treatment of RFA or surgical resection [6–9]. However, the use of RFA in the treatment of HCC is challenging when the tumor is located in the hepatic dome, near the macrovascular of liver, near the gallbladder, or near the gastrointestinal tract. For these patients, the radiotherapy might be a good choice. The efficacy comparison between the external stereotherapy and RFA in the treatment of patients with early stage HCC indicated that the median overall survival (OS) was similar [10]..

In recent years, the internal radiotherapy has been used to treat several types of tumors, and it was proved to be effective for HCC [11–14]. The internal radiotherapy of HCC mainly includes the implantation of iodine 125 seeds and transarterial radioembolization. The half-life of iodine 125 is 59.6 days, that ensures killing the tumor cells in a relatively long time. Iodine 125 radiates mostly X-rays and γ-rays to induce the mutations and/or damages of DNA in the tumor cells, and triggering apoptosis of the tumor cells [15]. Typically, in the treatment of HCC, the implantation of iodine 125 seeds is combined with other treatment modalities, such as RFA or surgery [16, 17]. It has been demonstrated that the combined treatment of TACE with iodine 125 seeds implantation (TACE-Iodine 125) provides a longer survival time than TACE alone for patients with HCC at different stages [18, 19]. However, the efficacy of TACE-Iodine 125 in early- and intermediate- stage HCC is still unclear. Moreover, there are no studies focusing on the comparison of the efficacy of TACE-Iodine 125 seeds implantation with TACE-RFA in patients with early- and intermediate-stage HCC. Thus, the objective of the present study was to estimate the efficacy of TACE-Iodine 125 and compare it with TACE-RFA in patients with early- and intermediate-stage HCC.

## Methods

### Study cohort

The medical records of consecutive 297 patients with HCC who received the treatment of RFA or iodine − 125 at our hospital between January 1, 2014 and May 31, 2018 were retrospectively reviewed. The inclusion criteria in this study were: (1) patients who were diagnosed with primary early-intermediate HCC by biopsy or imaging, based on European Association for the Study of the Liver (EASL) guideline and Barcelona Clinic Liver Cancer (BCLC) stage [2]; (2) patients received the treatment of TACE combined with iodine 125 or TACE combined with RFA; (3) patients with good liver function; (4) patients with the Eastern Cooperative Oncology Group (ECOG) score of 0; (5) patients with the platelet count higher than 40 × 10^9^/L. The exclusion criteria in this study were: (1) the tumors of patients invaded blood vessel or metastasis distant organs; (2) patients have received the treatments of TACE or RFA or iodine 125 or liver resection before been included into the study; (3) the tumors of patients were diffuse; (4) patients had a history of liver cancer rupture. According to the inclusion and exclusion criteria, a total of 112 patients were included in this study (Additional File Figure 1). Approval for this investigation was obtained from the Ethics Committee of our college Institutional Review Board, and the requirement for informed consent was waived.

The decision to perform the TACE-RFA or TACE-Iodine 125 was based on the multidisciplinary liver conference and patients’ preference prior to the operation. Patients with early stage HCC were recommended to receive surgical resection, liver transplantation, or RFA. Among them, some patients were not candidates for RFA treatment due to a suboptimal location of the tumor, and some declined the surgery because they had been treated surgically before inclusion in this study. For these patients, iodine 125 seeds were recommended as the first-line treatment. All patients with early stage HCC were recommended to receive the TACE treatment because of the available evidence that the efficacy of TACE-RFA or TACE-Iodine 125 therapies is better than a single treatment. Patients with intermediate stage HCC were recommended to receive TACE treatment. For these cases, RFA or iodine 125 seeds implantation acted as a adjuvant therapy because of the low tumor necrosis rate following the TACE treatment.

### Techniques

The extent of hepatic tumor burden was assessed before the surgery by triphasic dynamic enhanced computed tomography (CT) or magnetic resonance imaging (MRI) of liver. The liver function and patients’ medical condition were determined by laboratory tests and physical examinations, respectively.

### TACE procedure

The TACE was performed by two operators who respectively had at least 8 years and 20 years of experience in performing this type of procedures. Initially, the tip of a 5-French catheter (Cook, Bloomington, IN, USA) or 3-French microcatheter (Progreat, Terumo, Tokyo, Japan) was advanced into the tumor-feeding arteries. Then, an emulsion was prepared by mixing 1 part of lipiodol (Lipiodol Ultrafluido, Guerbet, Villepinte, France) and 2 parts of doxorubicin hydrochloride (Hisun Pharmaceutical Co. Ltd., Zhejiang, China). Depending on the liver function and the tumor size, 5-10 ml of the emulsion was injected through the catheter into the tumor-feeding arteries. Lastly, the embolization with gelatin sponge seeds (300–700 μm, Cook) was performed until the stasis of arteries flow was achieved.

### Iodine 125 seeds implantation

Iodine 125 seeds implantation was performed by three operators with seven, 10 and 15 years of experience in interventional radiology therapy, respectively. The iodine 125 seeds were implanted into the tumors under the guidance of ultrasound and CT imaging. The iodine 125 seeds were enclosed in the NiTinol capsule (China Institute of Atomic Energy, Beijing, China). The seeds with 0.8 mm in diameter and 4.5 mm in length were implanted into the tumors at 2–3 weeks after TACE. One week before the implantation, the patients underwent a CT scan of liver, and the CT images were transmitted to the Treatment Planning System (TPS). The number and positions of the iodine 125 seeds were determined by the TPS according to the minimum peripheral dose (mPD, 90 to 165Gy) prescribed for each tumor. Thus, X- and γ-rays could cover the planned target volume, including the tumor and 0.5–1 cm of adjacent non-tumorous tissue. The placement of the needles (18-gauge, XinKe Pharmaceutical Ltd., Shanghai, China) was performed under CT guidance, and the seeds were implanted into tumors at the interval of 1 to 1.5-cm through the needles. In the current study, a median of 20 seeds (range: 1–48 seeds) were implanted in each patient.

### Radiofrequency ablation

All the RFA procedures utilized the guidance of ultrasound and were conducted 1–2 weeks after the TACE procedure. The protocol was performed by two operators with 23 and 32 years of experience in interventional radiology, respectively. The tumor location was determined by ultrasound. Local lidocaine anesthesia was applied to relieve patients’ pain from puncture needles. Two grounding pads were attached to the patients’ legs. Subsequently, the electrode needles were inserted into the tumor under the guidance of the real-time ultrasound imaging. After the tips of the electrode needles were placed in the tumor, the 8 hook-shaped expandable probes of the electrode needle (Rita Medical System, Mountain View, CA, USA) were released to cover the entire tumor and 1-cm adjacent non-tumorous tissue. The ablation temperature was kept at 90–100 °C for 10–15 min. To ascertain that the ablation was complete, the patients received the enhanced CT scans to ensure no residual tumors. Otherwise, the RFA was repeated to achieve a complete ablation.

### Assessment of clinical outcomes and follow-up

The primary endpoint was overall survival (OS). The secondary endpoints were progress-free survival (PFS) and objective response rate (ORR). OS was defined as the time from the initial TACE procedure until the last follow-up or patient death. PFS was defined as the time from the first implantation of iodine 125 seeds or RFA treatment to the time of the diagnosis of tumor progression or patient death; this definition was based on the modified Response Evaluation Criteria in Solid Tumors (mRECIST) [20]. The ORR was defined as the percentage of patients with a response rated as a complete response (CR) and partial response (PR). CR was defined as no enhancement in the arterial stage, and PR was defined as 30% off of the treated tumor with a residual arterial enhancement. Tumor progression was defined as an increase in the size of the treated tumor by 20%, interval development of new intrahepatic tumors, or metastasis based on the mRECIST assessment.

All the patients underwent the follow-ups with laboratory and imaging examinations, and the end of the follow-up time was May 31, 2019. The median follow-up time was 29 months (range: 5–63 months). The patients were evaluated 1 month after initial treatment and then every 2 months by laboratory tests, contrast-enhanced CT, or contrast-enhanced MRI. The imaging results were evaluated by two radiologists and an interventional radiologist to decide whether the patients should receive a repeated treatment (TACE, RFA, or iodine 125 seeds implantation). The number of treatments for each patient was recorded.

### Statistical analysis

The preoperative characteristics of patients in the two groups were recorded and compared. Continuous variables were compared by a Student’s t-test or a Mann-Whitney U test, and categorical variables were analyzed by a Chi-square test or a Fisher’s exact test. The OS and PFS in the two groups and subgroups were calculated by the Kaplan-Meier method. A cox proportional risk model was used to analyze the predictors for death and recurrence, and adjuste the age, gender, Child-Pugh, BCLC stage and AFP. All the tests were two-tailed, and the *P*-value of less than 0.05 was considered statistically significant. SPSS 24.0 software (IBM, Chicago, IL, USA) and SAS software, version 9.4 (SAS Institute, Cary, NC) were used to perform the statistical analyses.

### Propensity score matching and inverse probability of treatment weighting

A propensity score matching (PSM) analysis was applied to reduce the selection bias and the potential confounding effects of this study. The following baseline characteristics of patients were included in the PSM assessment: age, gender, alanine aminotransferase (ALT), hemoglobin, platelet, lymphocyte, neutrophil, leukocyte, hepatitis B virus (HBV), alpha-fetoprotein (AFP) level, TACE number, number of tumors, tumor size level, Child-Pugh class, BCLC stage. A 1:1 ratio matching with an optimal caliper of 0.2 without replacement generated 35 pairs of patients. After the PSM, there was no significantly statistical difference of the baseline characteristics (Table 1).

**Table 1:** The baseline characteristics of patients before and after PSM

	Before PSM			After PSM		
Characteristics	TACE-Iodine 125 group	TACE-RFA group	*P* value	TACE-Iodine 125 group	TACE-RFA group	*P* value
Age (Years)	58.2 ± 9.7	57.7 ± 8.9	0.787	58.1 ± 10	57.6 ± 8.2	0.824
ALT (U/L)	39.9 ± 20.6	48.8 ± 40.9	0.211	39.7 ± 20.9	45.9 ± 27.6	0.287
Hemoglobin (g/L)	122.2 ± 22.1	127 ± 22.4	0.284	122.2 ± 23	127.4 ± 24	0.361
Platelet (×10^9^/L)	116 ± 58.5	131.1 ± 68.5	0.250	119 ± 59.7	138.7 ± 72.4	0.218
Lymphocyte(×10^9^/L)	1.2 ± 0.6	1.3 ± 0.6	0.786	1.2 ± 0.6	1.2 ± 0.6	0.911
Neutrophil (×10^9^/L)	3.2 ± 2.6	3.1 ± 1.7	0.712	3.3 ± 2.6	3.2 ± 1.9	0.939
Leukocyte (×10^9^/L)	5.2 ± 3.1	5 ± 2.3	0.763	5.2 ± 3.2	5.2 ± 2.5	0.937
Gender (male, %)	78.9	87.8	0.214	80	88.6	0.324
HBV(+, %)	63.2	75.7	0.165	65.7	68.6	0.799
AFP (>200μg/L, %)	21.1	23	0.817	22.9	34.3	0.290
TACE number (≥2, %)	89.5	73	0.044	88.6	82.9	0.495
Tumor number (≥2, %)	65.8	35.1	0.002	62.9	60	0.806
Tumor size (> 3 cm, %)	65.8	56.8	0.356	34.3	20	0.179
CHILD (B, %)	34.2	23	0.204	37.1	28.6	0.445
BCLC (B, %)	68.4	41.9	0.008	65.7	82.9	0.101

Abbreviations: *HBV* Hepatitis B Virus; *+* Positive, −, Other, *ALT* Alanine Aminotransferase; *AST* Aspartate Aminotransferase; *AFP* Alpha Fetoprotein; *BCLC* Barcelona Clinic Liver Cancer

Inverse probability weighting (IPTW) was used to estimate the average TACE-Iodine 125 treatment effect on the TACE-RFA treatment (TACE-Iodine 125 weight: [1 – propensity score]/propensity score; TACE-RFA weight: 1). After IPTW, there were 54.4 samples in the TACE-Iodine 125 group and 74 samples in the TACE-RFA group. The baseline characteristics of patients were presented in Additional File Table 1.

## Results

### Patients

From the total of 297 patients who were treated with RFA or iodine 125 seeds implantation, 112 patients met the inclusion criteria. Among them, 74 patient received the TACE-RFA treatment, and 38 received the TACE-iodine 125 seeds implantation treatment. The baseline preoperative characteristics of the patients are listed in Table 1. The mean age of all the patients was 57.9 years (range: 32–80 years); it averaged 57.5 years (range: 32–77 years) in the TACE-RFA group and 58.2 years (range: 33–80 years) in the TACE-Iodine 125 seeds implantation group. All the procedures were successfully performed in this study. In the TACE-RFA group, 69 patients received one RFA treatment, and 5 patients received two treatments. In the TACE-iodine 125 seeds implantation group, 25 patients received one iodine 125 seeds implantation treatment, 9 patients received two treatments, 2 patients received three treatments, and 2 patients received four treatments.

### Overall survival and progression free survival

**Fig. 1:**
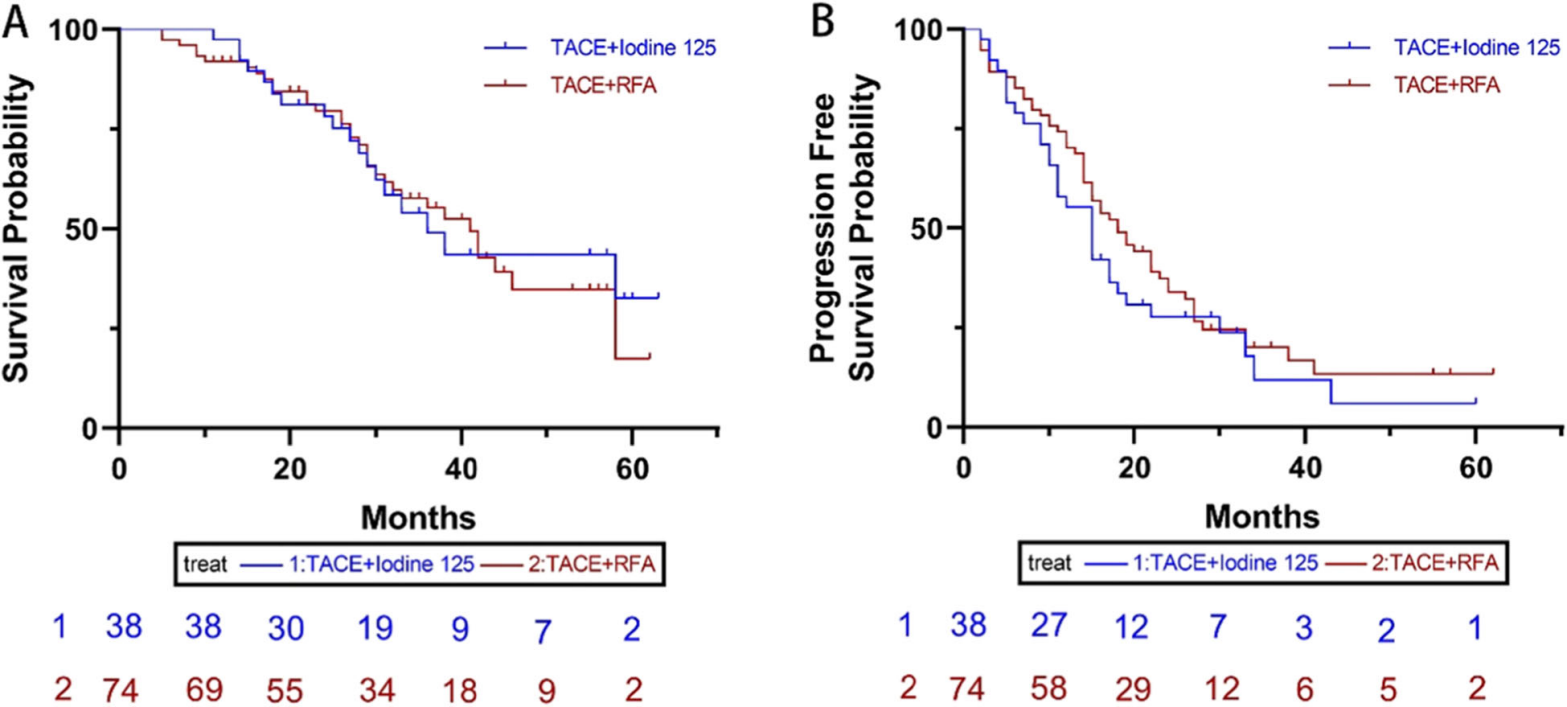
The Kaplan-Meier curve of overall survival (**a**) and progression free survival (**b**) of patients before PSM.

The data analysis before the PSM showed that 34 (45.9%) patients died in the TACE-RFA group, among them, 30 patients died of liver related reasons. In the TACE iodine 125 group, 18 (47.4%) patients died, among them, 16 patients died of liver related reasons. The median OS in the TACE-RFA group was 41 months (95%CI: 34.3, 47.7 months), which was slightly longer than that of in the TACE-Iodine 125 group (36 months, 95%CI: 27.2, 44.8 months). There was no statistically significant difference in the median OS between the two groups (*P* = 0.881) (Fig. 1a**)**. The median PFS was 18 months (95%CI: 13.3, 22.7 months) in the TACE-RFA group, and 15 months (95%CI: 11.4, 18.6 months) in the TACE-iodine 125 groups. No statistically significant difference was found between the two groups in the median PFS (*P* = 0.306) (Fig. 1b). The ORR was comparable between the two groups (67.6% in the TACE-RFA group, 73.7% in the TACE-Iodine 125 group, *P* = 0.505).

After adjusting the age, gender, Child-Pugh class, BCLC stage and AFP, level, the results of cox regression analysis for all patients before PSM determined that TACE-Iodine 125 treatment was not associated with the increased mortality (HR: 0.763; 95%CI: 0.403,1.345, *P* = 0.320) and recurrence (HR:1.020; 95%CI: 0.645,1.611, *P* = 0.934) (Table 2, Table 3).

**Table 2:** Associations between TACE-Iodine 125 treatment and mortality in patients with early-intermediate HCC before PSM

Characteristics	HR (95%CI)	*P* value	Adjusted HR (95%CI)	*P* value
**Overall**				
TACE-RFA	Reference		Reference	
TACE-Iodine 125	0.957 (0.538,1.704)	0.556	0.763 (0.403,1.345)	0.320
**Child-Pugh A**				
TACE-RFA	Reference		Reference	
TACE-Iodine 125	1.001 (0.513,1.953)	0.998	0.822 (0.414,1.633)	0.576
**Child-Pugh B**				
TACE-RFA	Reference		Reference	
TACE-Iodine 125	1.095 (0.331,3.621)	0.882	0.538 (0.124,2.342)	0.409
**BCLC A**				
TACE-RFA	Reference		Reference	
TACE-Iodine 125	0.854 (0.242,3.009)	0.806	0.496 (0.125,1.973)	0.319
**BCLC B**				
TACE-RFA	Reference		Reference	
TACE-Iodine	0.826 (0.425,1.606)	0.573	0.834 (0.422,1.650)	0.603

Abbreviations: *TACE* Transarterial chemoembolization; *RFA* Radiofrequency ablation; *BCLC* Barcelona Clinic Liver Cancer

**Table 3:** Associations between TACE-Iodine 125 treatment and recurrence in patients with early-intermediate HCC before PSM

Characteristics	HR (95%CI)	*P* value	Adjusted HR (95%CI)	*P* value
**Overall**				
TACE-RFA	Reference		Reference	
TACE-Iodine 125	1.252 (0.805,1.950)	0.319	1.020 (0.645,1.611)	0.934
**Child-Pugh A**				
TACE-RFA	Reference		Reference	
TACE-Iodine 125	1.270 (0.749,2.154)	0.374	0.976 (0.566,1.685)	0.932
**Child-Pugh B**				
TACE-RFA	Reference		Reference	
TACE-Iodine 125	1.416 (0.598,3.354)	0.429	1.539 (0.560,4.232)	0.403
**BCLC A**				
TACE-RFA	Reference		Reference	
TACE-Iodine 125	0.816 (0.352,1.893)	0.636	0.601 (0.234,1.547)	0.291
**BCLC B**				
TACE-RFA	Reference		Reference	
TACE-Iodine	1.253 (0.724,2.169)	0.421	1.252 (0.722,2.171)	0.423

Abbreviations: *TACE* Transarterial chemoembolization; *RFA* Radiofrequency ablation; *BCLC* Barcelona Clinic Liver Cancer

**Fig. 2:**
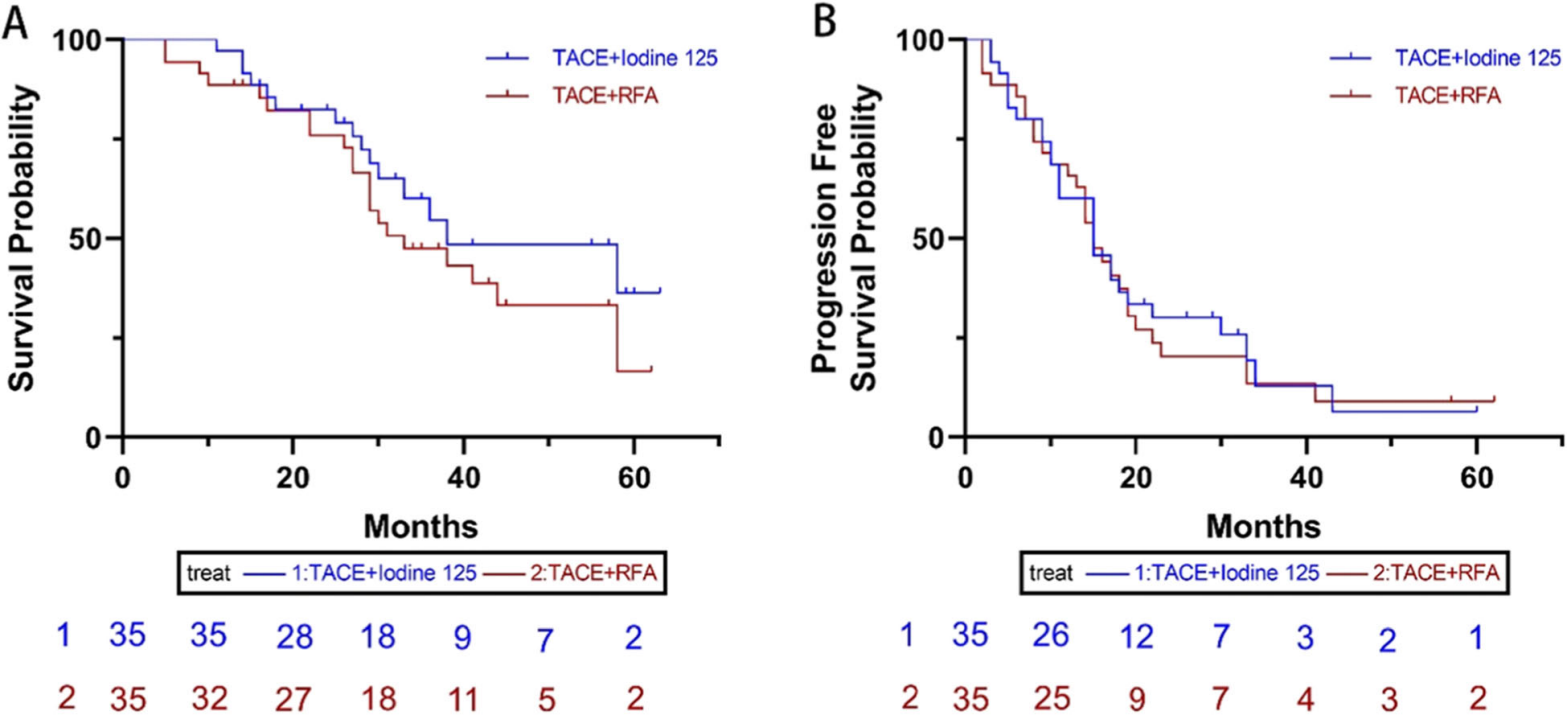
The Kaplan-Meier curve of overall survival (**a**) and progression free survival (**b**) of patients after PSM.

After the PSM protocol, 35 patient pairs were identified. The median OS in the TACE-RFA group was 33 months (95%CI: 21.7, 44.3 months), which was slightly shorter than that of in the TACE-Iodine 125 group (38 months, 95%CI: 16.2, 44.3 months); this difference between the two groups did not reach the statistically significant difference (*P* = 0.279) (Fig. 2a). Meanwhile, no statistically significant difference of the median PFS was present between the two groups (TACE-RFA: 15 months, 95%CI: 12.3, 17.7 months, and TACE-Iodine 125 seeds implantation: 15 months, 95%CI: 10.1, 19.9 months; *P* = 0.790) (Fig. 2b). The ORR did not differ statistical significantly (*P* = 0.131) between the TACE-RFA group (57.5%, 20 of 35 pairs) and the TACE-Iodine 125 group (74.2%, 26 of 35 pairs). The cox regression analysis of all patients showed that the TACE-Iodine 125 treatment was not associated with the increased mortality (HR:0.766; 95%CI: 0.384,1.530, *P* = 0.450) and recurrence (HR:1.220; 95%CI: 0.711,2.093, *P* = 0.470) (Table 4).

**Table 4:** Associations between TACE-Iodine 125 treatment and mortality and recurrence in patients with early-intermediate HCC after PSM

Characteristics	Mortality		Recurrence	
	Adjusted HR (95%CI)	P value	Adjusted HR (95%CI)	*P* value
**Overall**				
TACE-RFA	Reference		Reference	
TACE-Iodine 125	0.766 (0.384,1.530)	0.450	1.220 (0.711,2.093)	0.470

Abbreviations: *TACE* Transarterial chemoembolization; *RFA* Radiofrequency ablation; *BCLC* Barcelona Clinic Liver Cancer

After IPTW, there was no statistically significant difference of mOS (41 months vs N, *P* = 0.230) and mPFS (18 months vs 19 months, *P* = 0.358) between the TACE-RFA group and the TACE-Iodine 125 group (Additional File Figure 2). The ORR between the TACE-RFA group and TACE-Iodine 125 group was similar (67.6% vs 77.9%, *P* = 0.196). The cox regression analysis showed that TACE-Iodine 125 might decrease the mortality (HR: 0.495, 95%CI: 0.280,0.874, *P* = 0.015), but not the tumor recurrence (HR: 0.724, 95%CI: 0.472,1.110, *P* = 0.128) compared with TACE-RFA (Additional File Table 2).

The subgroup analysis of BCLC stage A and BCLC stage B patients between the two groups showed that there was no statistically significant difference of mOS and mPFS between the two groups (all *P* > 0.05) (Additional File Table 3 and Additional File Figure 3-5)

### Complications

There were no procedure-related death and no iodine 125 particle migration from the tumor to the lung, heart, or other organs. One patient died of liver failure at 2 days after receiving the third treatment with iodine 125. After PSM, the leucopenia and increased ALT were significantly worse in the TACE-iodine 125 group than that of in the TACE-RFA group. The fever, liver pain, nausea, and fatigue were typically occurred at 1 to 7 days after the procedures and were relieved at 1–2 weeks after receiving the symptoms treatment (Table 5).

**Table 5:** Adverse events of patients with TACE-Iodine 125 or TACE-RFA treatments after PSM

Adverse events	Grade I and II			Grade III and IV		
	TACE-RFA	TACE-Iodine 125	*P* value	TACE-RFA	TACE-Iodine 125	*P* value
Fatigue	10	12	0.607	1	1	> 0.99
Nausea	8	8	> 0.99	0	1	> 0.99
Fever	3	7	**0.172**	0	0	> 0.99
Leucopenia	0	6	**0.003**	0	0	> 0.99
Liver pain	25	24	0.794	2	2	> 0.99
Hepatic hemorrhage	3	2	0.642	1	0	> 0.99
Increased ALT	6	14	**0.034**	3	6	0.280
Pneumothorax	2	4	0.389	0	0	> 0.99

Abbreviations: *TACE* Transarterial chemoembolization; *RFA* Radiofrequency ablation;

## Discussion

In our institution, surgery and RFA are recommended as the first-line treatment for patients with early stage of HCC, and TACE is recommended as the first-line treatment for patients with intermediate stage HCC. The combination of TACE and RFA was deemed appropriate for the patients with early stage HCC since the previous studies had demonstrated the better outcomes in the patients receiving the combined therapies than in those subjecting to a single treatment [21, 22]. The iodine 125 seeds implantation was used as a treatment option for the patients who were not the candidates for RFA or surgery, or for the patients who were willing to receive the treatment due to its low cost and risk. At present, it is well-established that the combination of TACE with iodine 125 is more effective than TACE alone in the treatment of HCC. However, it remains unknown that whether iodine 125 is more beneficial than RFA for these patients. Thus, the purpose of this study was to assess the efficacy of TACE-Iodine 125 seeds implantation and compare it with TACE-RFA for the patients with HCC who were unfit or unwilling to undergo the TACE-RFA treatment.

The implantation of iodine 125 seeds has been used in the treatment of HCC, and its therapeutic efficacy was encouraging. The advantage of this procedure in the treatment of tumors is that it rarely damages the surrounding normal tissue for its limited radiation distance. A previous study, that compared the efficacy of RFA-alone treatment and the combined treatment of RFA with iodine 125 seeds implantation for the patients with early stage of HCC, demonstrated that the RFA-Iodine 125 group had a longer survival time than RFA-alone group [16]. Additional studies compared the effects of TACE-Iodine 125 seeds implantation with those of TACE alone in the treatment of HCC based on BCLC stage or tumor size; the results indicated the better outcomes in the patients who were treated with the combined therapy [18, 23, 24]. The current study did not identify the statistically significant differences in the OS and PFS between the two groups. The ORR was similar between the TACE-RFA group and the TACE-Iodine 125 seeds implantation group. The previous studies implied that BCLC stage, AFP level, Child-Pugh class, and among others, might influence the outcomes of HCC patients who were treated with various therapies. Hence, the patients who received the treatment of TACE-RFA or TACE-Iodine 125 in the present study were compared based on liver resection, BCLC stage, AFP level, and Child-Pugh class. The results indicated that TACE-Iodine 125 treatment didn’t significantly increase the mortality and recurrence compared with TACE-RFA treatment. To reduce the impact of selective bias on the comparison of the therapeutic efficacy between the two groups, a PSM analysis and the method of IPTW were performed. The matching factors that were used for PSM analysis included the tumor burden, liver function, physical capacity, and risk factors for patients with HCC. After PSM and IPTW, there were no statistically significant differences between the two groups in the OS, PFS, and ORR.

The concerns regarding the possibility of damaging organs surrounding the tumor by X-ray and γ-ray and bleeding resulting from the insertion of needles into a vascularized organ have limited the use of implantation of iodine 125 seeds in HCC treatment. However, in this study, only two patients suffered from hepatic hemorrhage in the TACE-iodine 125 group, and migration of particles to other organs was not observed. However, the postoperative increased ALT, and leucopenia were more frequent in the patients who were treated with TACE-Iodine 125 than that of in the TACE-RFA group. The symptoms were relieved by the symptomatic treatment. Thus, the TACE-Iodine 125 treatment appeared as safe as the TACE-RFA treatment. Furthermore, in China, the cost of once RFA treatment is approximately $2800–$3500 and one iodine 125 seed is about $58. In this study, the median iodine 125 seeds were 20 (1–48), so the cost of once iodine 125 seeds implantation usually does not exceed $2000, which is lower than RFA. The number of iodine 125 seeds implantation is similar with RFA in this study (median: 1 vs 1), which showed that iodine 125 might be a cost effective method in the treatment of early-intermediate HCC.

Although this study provided the encouraging results on the TACE-Iodine 125 seeds implantation treatment in patients with early- and intermediate-stage HCC compared with the TACE-RFA treatment, some limitations have to be acknowledged. First, despite the implementation of PSM, an inherent selection bias might be present due to the retrospective character of the study. Second, the follow-up time was not long, which might lead to an underestimation of OS and PFS. However, the obtained results were encouraging and documented the feasibility and efficacy of a novel selective treatment for the patients with early- and intermediate-stage HCC.

## Conclusions

In conclusion, the combined treatment of TACE with implantation of iodine 125 seeds may be an effective and safe alternative therapy for patients with early- and intermediate-stage HCC who cannot undergo RFA or surgery treatment.

## Supplementary information

**Additional file 1 Table S1**: The baseline characteristics of patients after IPTW. **Table S2**: Associations between TACE-Iodine 125 treatment and mortality and recurrence in patients with early-intermediate HCC after IPTW. **Table S3**: Comparison the mOS and mPFS of BCLC A and B patients between TACE-Iodine 125 group and TACE-RFA group before and after PSM and after IPTW. **Figure S1**: The flowchart of patients inclusion. **Figure S2**: The Kaplan-Meier curve of overall survival (A) and progression free survival (B) of patients after IPTW. **Figure S3**: The Kaplan-Meier curve of overall survival and progression free survival of BCLC A (A-B) and BCLC B (C-D) patients before PSM. **Figure S4**: The Kaplan-Meier curve of overall survival and progression free survival of BCLC A (A-B) and BCLC B (C-D) patients after PSM. **Figure S5**: The Kaplan-Meier curve of overall survival and progression free survival of BCLC A (A-B) and BCLC B (C-D) patients after IPTW
