# Supplementary material for: Transarterial chemoembolization combined with iodine 125 seeds versus transarterial chemoembolization combined with radiofrequency ablation in the treatment of early- and intermediate-stage hepatocellular carcinoma
Source: BMC Gastroenterol. 2020 Jun 29;20:205. doi: 10.1186/s12876-020-01355-3 (PMC7324959; doi:10.1186/s12876-020-01355-3)
Supplement: Supplementary file 1 — Additional file 1 Table S1: The baseline characteristics of patients after IPTW. Table S2: Associations between TACE-Iodine 125 treatment and mortality and recurrence in patients with early-intermediate HCC after IPTW. Table S3: Comparison the mOS and mPFS of BCLC A and B patients between TACE-Iodine 125 group and TACE-RFA group before and after PSM and after IPTW. Figure S1: The flowchart of patients inclusion. Figure S2: The Kaplan-Meier curve of overall survival (A) and progression free survival (B) of patients after IPTW. Figure S3: The Kaplan-Meier curve of overall survival and progression free survival of BCLC A (A-B) and BCLC B (C-D) patients before PSM. Figure S4: The Kaplan-Meier curve of overall survival and progression free survival of BCLC A (A-B) and BCLC B (C-D) patients after PSM. Figure S5: The Kaplan-Meier curve of overall survival and progression free survival of BCLC A (A-B) and BCLC B (C-D) patients after IPTW [file 12876_2020_1355_MOESM1_ESM.docx]

**Table 1: The baseline characteristics of patients after IPTW**

| Characteristics | TACE+Iodine 125  n=54.44 | TACE+RFA  n=74 | P value |
| --- | --- | --- | --- |
| Age (Years) | 57.2±12.6 | 57.7±8.9 | 0.794 |
| ALT (U/L) | 40.1±24.7 | 48.8±40.9 | 0.141 |
| Hemoglobin (g/L) | 122.6±26.9 | 127±22.4 | 0.304 |
| Platelet (×10^9^/L) | 137.1±78.3 | 131.1±68.5 | 0.641 |
| Lymphocyte(×10^9^/L) | 1.2±0.6 | 1. 3±0.62 | 0.453 |
| Neutrophil (×10^9^/L) | 3±2.3 | 3.1±1.7 | 0.883 |
| Neutrophil (×10^9^/L) | 4.9±3.1 | 5±2.3 | 0.782 |
| Gender (Male, %) | 80.90 | 87.8 | 0.278 |
| HBV (+, %) | 72.42 | 75.7 | 0.676 |
| AFP (>200ug/L, %) | 24.02 | 23 | 0.890 |
| TACE number (≥2, %) | 89.41 | 73 | 0.022 |
| Tumor number (≥2, %) | 46.82 | 35.1 | 0.182 |
| Tumor size (>3cm, %) | 63.98 | 56.8 | 0.409 |
| CHILD (B, %) | 27.72 | 23 | 0.540 |
| BCLC (B, %) | 53.8 | 41.9 | 0.183 |

Abbreviations: HBV, Hepatitis B Virus; +, Positive, -, Other, ALT, Alanine Aminotransferase; AST, Aspartate Aminotransferase; AFP, Alpha Fetoprotein; BCLC, Barcelona Clinic Liver Cancer

**Table 2: Associations between TACE-Iodine 125 treatment and mortality and recurrence in patients with early-intermediate HCC after IPTW**

| Characteristics | Mortality | | Recurrence | |
| --- | --- | --- | --- | --- |
|  | Adjusted HR (95%CI) | P value | Adjusted HR (95%CI) | P value |
| **Overall** |  |  |  |  |
| TACE-RFA | Reference |  | Reference |  |
| TACE-Iodine 125 | 0.495 (0.280,0.874) | 0.015 | 0.724 (0.472,1.110) | 0.138 |

Abbreviations: TACE, Transarterial chemoembolization; RFA, Radiofrequency ablation; BCLC, Barcelona Clinic Liver Cancer.

**Table 3: Comparison the mOS and mPFS of BCLC A and B patients between TACE-Iodine 125 group and TACE-RFA group before and after PSM and after IPTW.**

|  | mOS (Months) | | | | | | mPFS (Months) | | | | | |
| --- | --- | --- | --- | --- | --- | --- | --- | --- | --- | --- | --- | --- |
|  | BCLC A | | | BCLC B | | | BCLC A | | | BCLC B | | |
|  | TACE+  Iodine 125 | TACE+RFA | P value | TACE+  Iodine 125 | TACE+RFA | P value | TACE+ Iodine 125 | TACE+RFA | P value | TACE+ Iodine 125 | TACE+RFA | P value |
| Before PSM | **N** | 44 | 0.805 | 36 | 30 | 0.565 | 33 | 24 | 0.630 | 11 | 14 | 0.405 |
| After PSM | **N** | **N** | 0.459 | 38 | 30 | 0.313 | 33 | 33 | 0.532 | 11 | 14 | 0.603 |
| After IPTW | **N** | 44 | 0.251 | 38 | 30 | 0.464 | 43 | 24 | 0.128 | 12 | 14 | 0.618 |

Abbreviations: OS, Overall survival; PFS, Progress Free Survival; TACE, Transarterial chemoembolization; RFA, Radiofrequency ablation; BCLC, Barcelona Clinic Liver Cancer; **N**, The exact survival time was not available.

**Figure 1: The flowchart of patients inclusion**

**
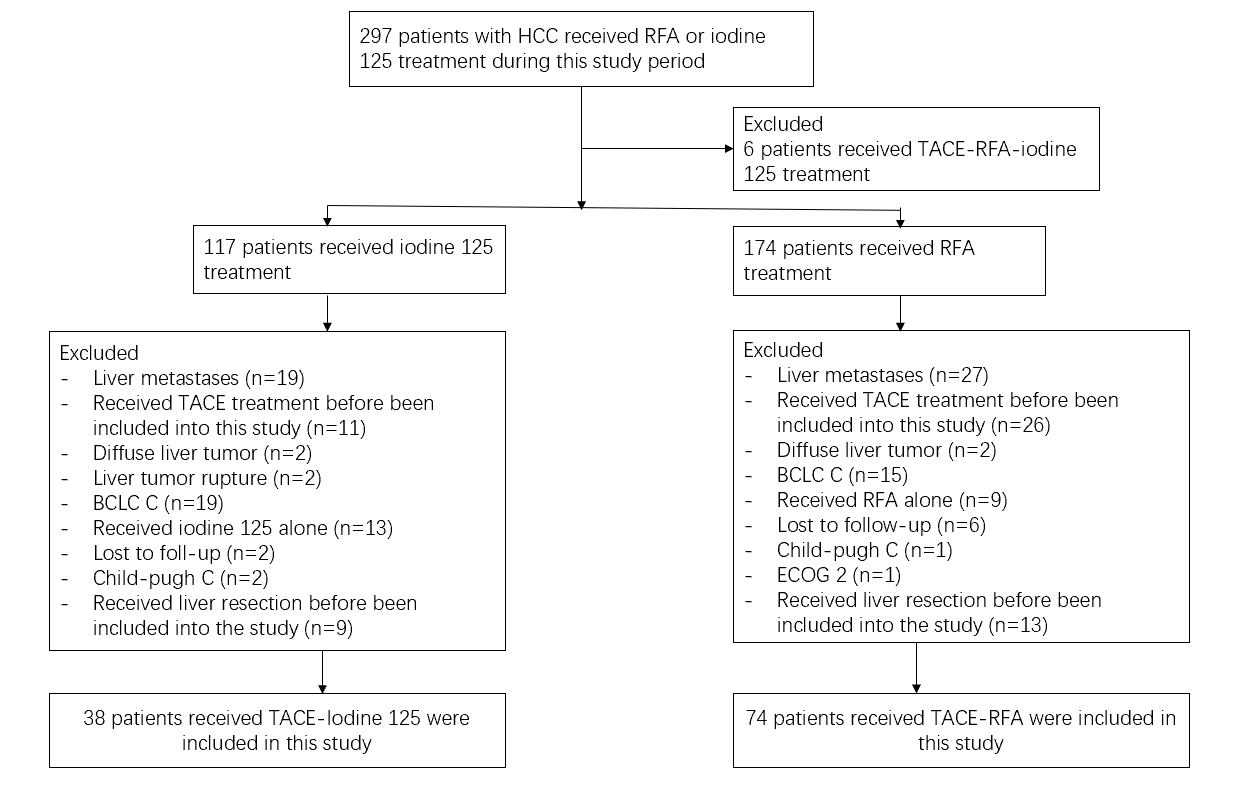
**

**Figure 2: The Kaplan-Meier curve of overall survival (A) and progression free survival (B) of patients after IPTW**

**
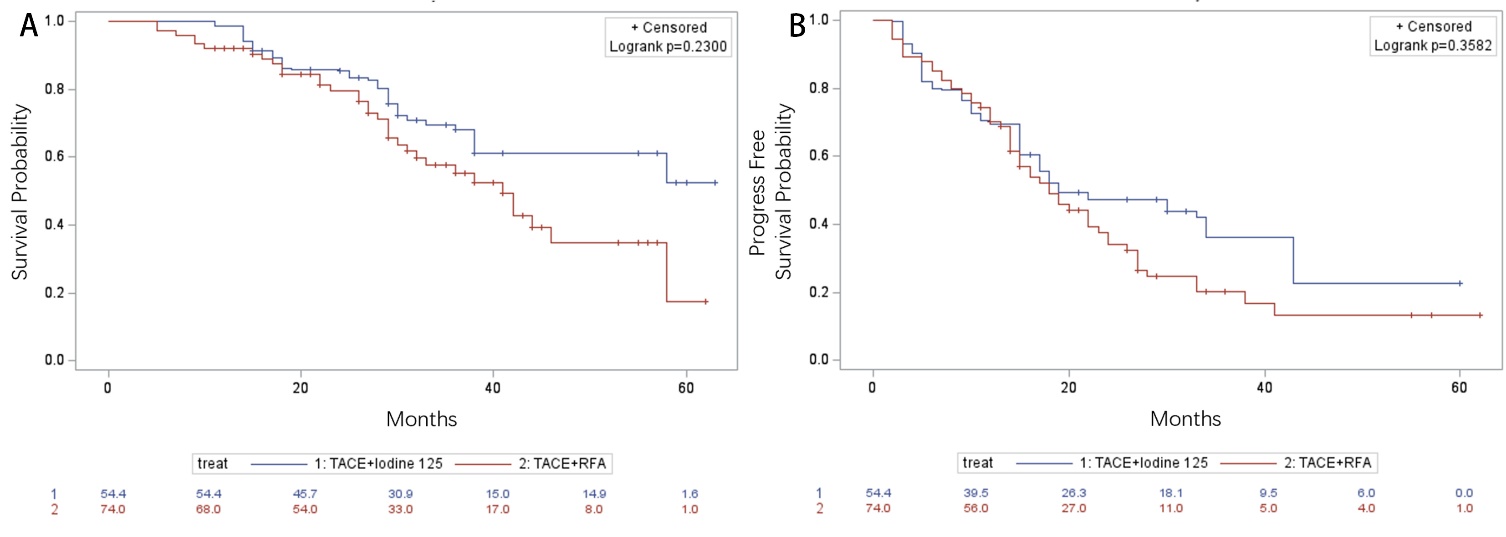
**

**Figure 3: The Kaplan-Meier curve of overall survival and progression free survival of BCLC A (A-B) and BCLC B (C-D) patients before PSM**

**
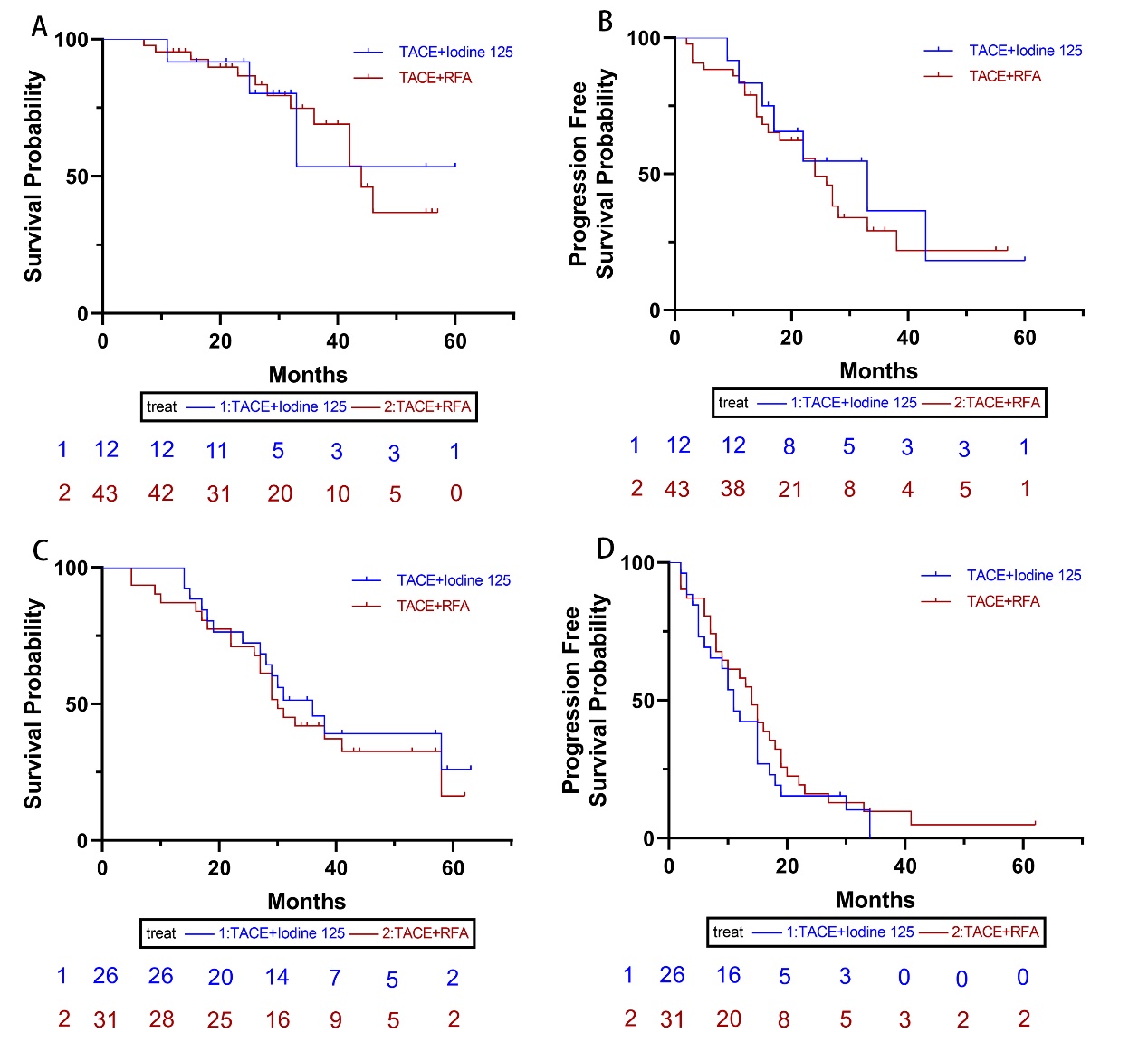
**

**Figure 4: The Kaplan-Meier curve of overall survival and progression free survival of BCLC A (A-B) and BCLC B (C-D) patients after PSM**

**
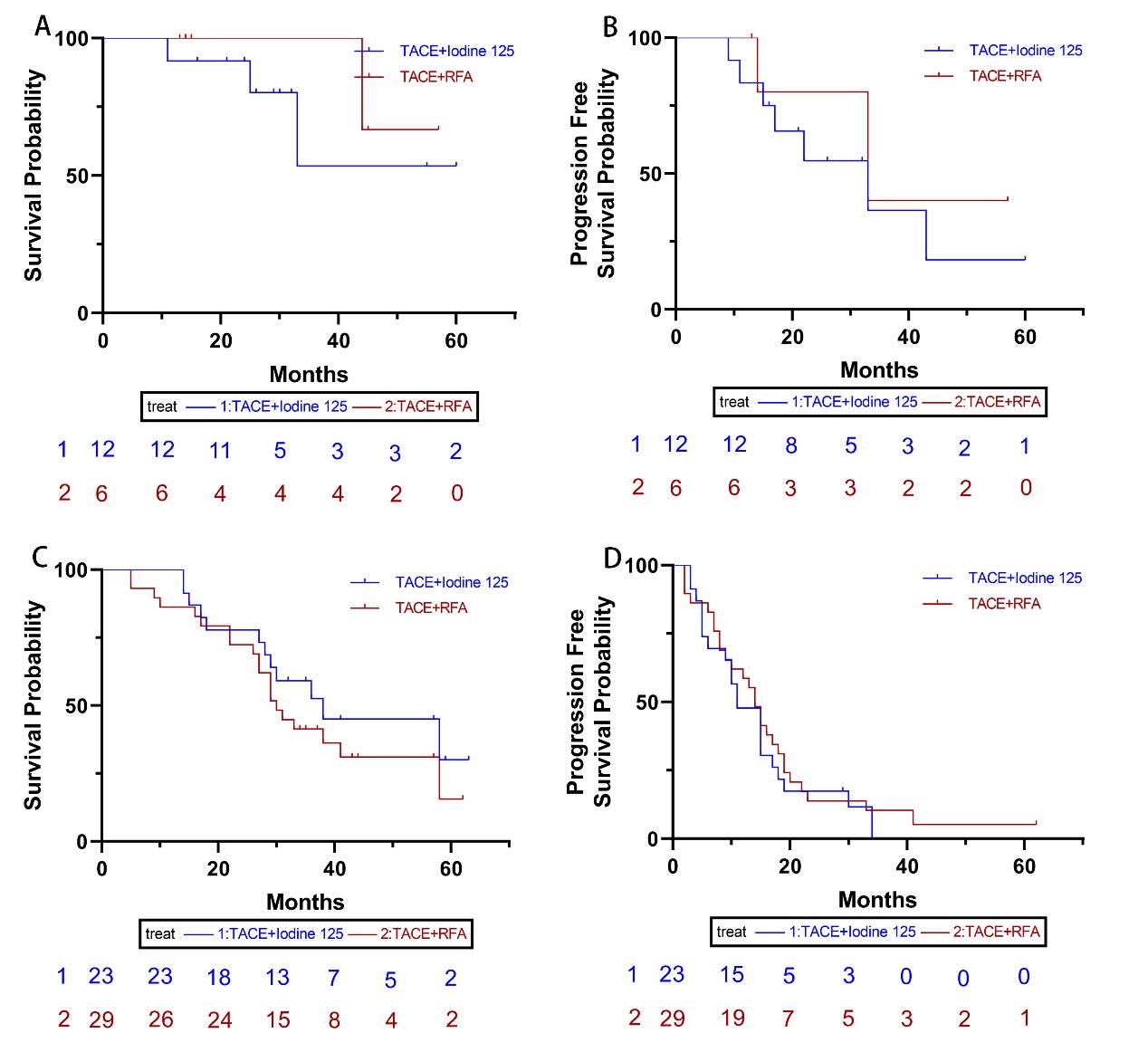
**

**Figure 5: The Kaplan-Meier curve of overall survival and progression free survival of BCLC A (A-B) and BCLC B (C-D) patients after IPTW**

**
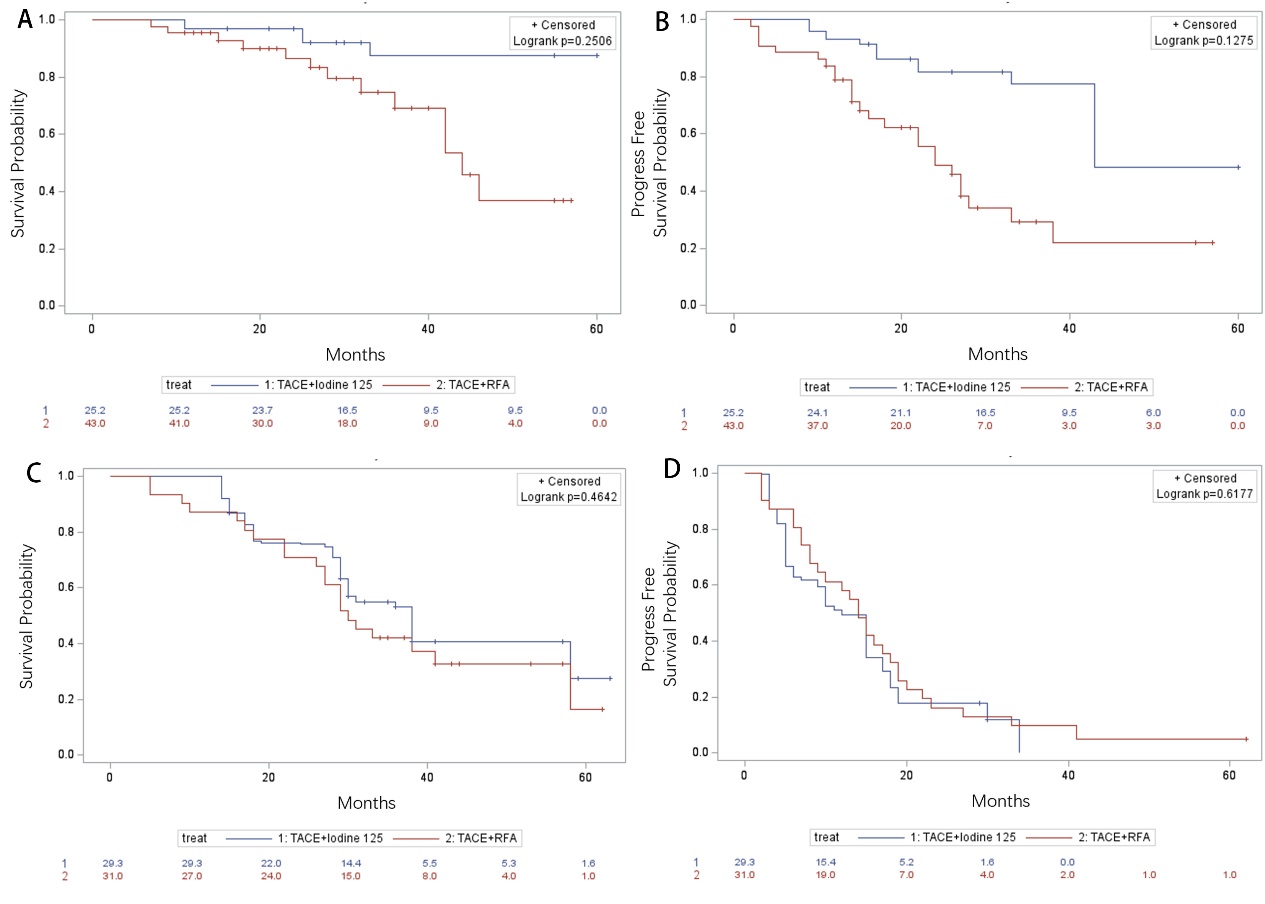
**
